# Gel-Based Marangoni Actuators: Mechanisms, Material Designs, Driving Modes, and Cross-Scale Applications

**DOI:** 10.3390/gels11090730

**Published:** 2025-09-11

**Authors:** Xuehao Feng, Zhizheng Gao, Wenguang Yang, Shuliang Zhu

**Affiliations:** School of Electromechanical and Automotive Engineering, Yantai University, Yantai 264005, China; 17660278786@163.com (X.F.); gaozhizheng2024@163.com (Z.G.)

**Keywords:** Marangoni effect, Marangoni actuators, interfacial tension gradients, actuation mechanisms, cross-scale applications

## Abstract

Marangoni actuators, rooted in interfacial tension gradients, stand as a significant advancement in micro-nano engineering. This review synthesizes their core mechanisms, which hinge on establishing gradients via temperature or solute concentration, with structural designs facilitating directional motion. Key actuation modalities, encompassing light, chemical, and electric driving, exhibit distinct characteristics in controllability and responsiveness. Their applications span cross-scale scenarios, from microscopic operations to macroscopic functional implementations. Current challenges involve optimizing performance and enhancing multi-field coordination, while future directions focus on advanced materials, intelligent regulation, and scalable fabrication. These actuators hold substantial potential in interdisciplinary fields, such as biomedicine, environmental engineering, and microfluidics.

## 1. Introduction

**Figure 1:**
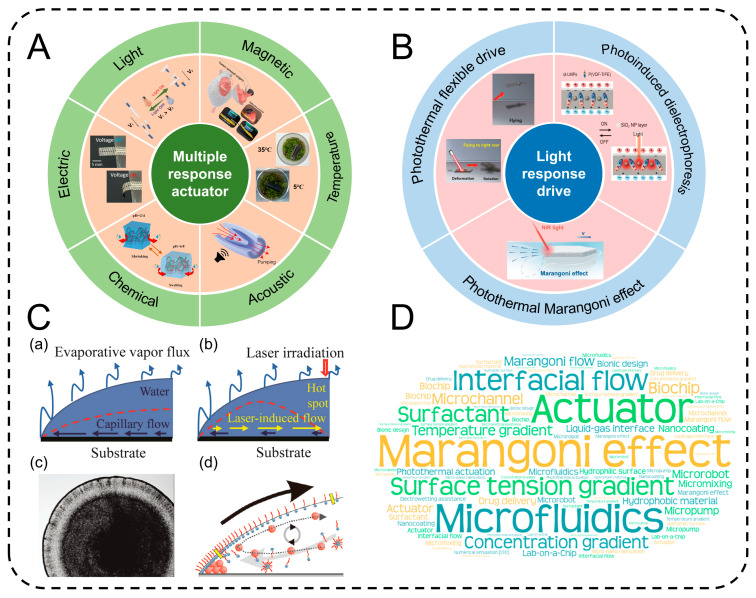
Mechanism analysis and research landscape visualization of multi-field regulated intelligent actuators. (**A**) External field driving paradigms of multi-responsive intelligent actuators. Light-driven. Reproduced from Reference [28] with permission from *Nature Communications*. Magnetic-driven. Reproduced from Reference [5] with permission from *Science Advances*. Temperature-driven. Reproduced from Reference [6] with permission from *Carbon*. Acoustic-driven. Reproduced from Reference [8] with permission from *Advanced Science*. Chemical-driven. Reproduced from Reference [9] with permission from *Carbohydrate Polymers*. Electric-driven. Reproduced from Reference [7] with permission from *Science Advances*. (**B**) Schematic diagram of the subdivision mechanism driven by light response. Photothermal flexible drive. Reproduced from Reference [10] with permission from *Nature Communication*. Photoinduced dielectrophoresis. Reproduced from Reference [11] with permission from *National Science Review*. Photothermal Marangoni effect. Reproduced from Reference [12] with permission from *Advanced Materials*. (**C**) Schematic illustration of thermal and solutal Marangoni flow directions. (**a**) Schematic of thermal-gradient-induced Marangoni flow during natural evaporation. (**b**) Regulation of hot-spot flow field induced by laser irradiation. Reproduced from Reference [29] with permission from *Soft Matter*. (**c**) Evaporation morphology characterization. (**d**) Solutal-gradient-driven Marangoni flow behavior. Reproduced from Reference [30] with permission from *Langmuir*. (**D**) Keyword cloud map of micro-nano driver research based on Marangoni effect.

Against the backdrop of the rapid evolution of advanced micro-nano robotics technology, various outfield-driven intelligent actuators have demonstrated broad application prospects [1,2,3,4]. Multifunctional responsive actuators driven by magnetic fields [5], thermal fields [6], electric fields [7], sound fields [8], and chemical fields [9] are gradually emerging in many cutting-edge fields. These external field-driven modes, through synergistic coupling with material properties, endow robots with diverse motion behaviors and functional realization capabilities, significantly expanding the application boundaries of micro-nano robots (Figure 1A).

It is worth noting that light, as a clean and highly controllable external field, has attracted much attention in the field of micro-nano robot driving. Light response drive, with its unique advantages, such as being non-contact and having high spatiotemporal resolution, has injected new impetus into the development of micro-nano robots. Optical drive mainly includes modes like photothermal flexible drive [10], photoinduced dielectrophoresis drive [11], and photothermal Marangoni effect drive [12]. The photothermal flexible drive induces thermal deformation of materials through photothermal effects, enabling the robot to perform movements like flight and deformation [13]. Light-induced dielectrophoretic drive utilizes the dielectric property differences caused by light to generate dielectrophoretic forces, achieving precise manipulation of micro-nano structures [14]. The photothermal Marangoni effect drive is unique. It builds a temperature gradient through light irradiation and then induces the formation of a surface tension gradient [15]. This difference in surface tension caused by the photothermal effect provides an efficient driving force for the surface swimming actuator. Compared with other light-driven methods, the photothermal Marangoni effect drive enables the actuator to achieve high-precision and high-efficiency movement on the liquid surface, demonstrating irreplaceable application potential in cutting-edge fields, such as microfluidic control, biomedical precision diagnosis and treatment, and in situ environmental monitoring. It is becoming a highly promising research direction in the field of surface swimming actuators (Figure 1B).

Traditional actuators mostly rely on mechanical transmission mechanisms, such as motors and hydraulics, which have structural redundancy, large volume, and miniaturization bottlenecks. Moreover, rigid components significantly limit the flexibility of movement. Meanwhile, physical contact or wired power supply can easily interfere with the working environment. In special scenarios, such as liquids and organisms, it also faces issues like wear and biocompatibility. Its motion control is also limited by the preset structure, and its adaptability to complex environments is relatively weak [16]. In contrast, the Marangoni actuator relies on the external field to regulate the surface tension gradient for driving without the need for complex mechanical components. It has a streamlined structure and is easy to miniaturize, allowing it to perform operations deep in microscopic spaces. Its motion modes are flexible and diverse. By adjusting the external field parameters, complex trajectory planning can be achieved, and it supports remote contactless control with minimal environmental interference. It can effectively adapt to sensitive scenarios, such as biomedicine and microfluidics [17]. In addition, this type of actuator has a natural compatibility with liquid environments, high motion efficiency, and a wide range of material options. It can be designed as a flexible and biosafe device and has significant advantages in targeted drug delivery and other fields. Its core characteristics of “replacing hardness with softness” and “interface energy driving” effectively break through the inherent bottlenecks of traditional driving technologies [18].

As a versatile class of soft-matter carriers, hydrogels, leveraging their inherent physicochemical properties, have emerged as the core building blocks in Marangoni actuators. They can effectively serve as structural scaffolds, fuel reservoirs, and responsive elements [19]. Their excellent mechanical deformation characteristics enable them to mediate adaptive interfacial interactions and achieve structural compatibility with dynamic flow fields, thereby providing support for efficient energy conversion at fluid interfaces [20]. Moreover, the inherent biocompatibility of hydrogels endows them with natural applicability in the biomedical field. In this domain, compatibility with the physiological microenvironment is crucial for applications like targeted cargo delivery and in situ monitoring [21]. The unique controllable swelling–deswelling kinetics and porous network structure of hydrogels allow them to encapsulate solutes (such as surfactants or chemical fuels) and achieve precise spatiotemporal controlled release. This provides essential support for the establishment of the solute concentration gradients on which Marangoni-driven motion relies [22]. Furthermore, their tunable response characteristics to external stimuli (such as light, temperature, or pH) can form synergistic coupling with the Marangoni effect, offering the possibility for precise regulation of driving behavior and motion control. These comprehensive properties collectively establish hydrogels as an ideal material system for driving the design innovation and functional expansion of Marangoni actuators.

From the perspective of the intersection of scientific mechanisms and application exploration, the unique value driven by the Marangoni effect is not only reflected in the technical implementation level, but also in providing a brand-new paradigm for cross-scale interface regulation [23]. Unlike the limitations of a single physical field-driven effect, the Marangoni effect can construct a dynamically tunable energy gradient field at the microscopic interface by coupling multiple physical and chemical parameters such as temperature, concentration, and electric field. This multi-field coupling characteristic enables precise force-motion conversion at the nanometer to centimeter scale [19,24,25]. For instance, at the microscopic scale, by regulating the photothermal power, the continuous adjustable driving force at the piconton level can be achieved, making it possible for fine operations, such as single-cell manipulation [26]. At the macroscopic scale, by leveraging the synergistic effect of large-area surface tension gradients, efficient transportation of gram-level loads can be achieved. This cross-scale regulation capability is unmatched by other interfacial driving mechanisms [27].

This article, as a functional piece, provides a comprehensive overview of the Marangoni actuator, with a particular focus on elaborating the driving mechanism behind the Marangoni effect and its material structure design, as well as the driving mechanisms and practical applications for different application scenarios. The article begins with a brief introduction to the various mechanisms that can currently generate the Marangoni flow and the responsive materials and structural designs adopted based on different mechanisms and then deeply analyzes their respective advantages. Subsequently, various mechanisms that can currently generate the Marangoni flow are described. This includes thermal-field-driven, electric-field-driven, concentration-field-driven, etc., laying a theoretical foundation for the subsequent functional design of Marangoni actuators. Then, while analyzing the potential development directions, the practical applications of the Marangoni actuator are introduced. At the micro level, it covers microfluidic manipulation, microscopic particle capture, etc. At the macro level, it involves fields like macroscopic supramolecular assembly, cargo transportation, programmable motion control, power generation, targeted drug delivery, cleaning, and sensing. Finally, in combination with existing research progress, the current challenges are pointed out, and the future development trends and prospects of this field are described.

## 2. Marangoni Actuators: In-Depth Analysis of Principle, Materials, and Structure

### 2.1. The Motion Principle of the Marangoni Actuator

The Marangoni effect was first systematically proposed by the Italian physicist Carlo Marangoni in 1865. It is a type of interfacial fluid dynamics phenomenon dominated by the surface tension gradient in liquid systems. It plays a key role in the mass transfer, heat transfer, and momentum transport processes of multiphase fluid systems [31]. The camphor boats and soap-driven devices from the 19th to 20th centuries intuitively demonstrated the interaction between interfacial tension gradients and directional motion, laying the conceptual foundation for the design of modern Marangoni actuators [32]. Its essential feature is manifested as follows. At interfaces, such as liquid–gas, liquid–liquid, or liquid–solid, and within droplets, the spatial non-uniform distribution of surface tension forms a continuous gradient field. This gradient field drives the fluid to undergo directional macroscopic migration through the coupling effect of interfacial shear forces. Specifically, it is manifested as the flow from the low surface tension area to the high surface tension area, and eventually a stable spontaneous convection system is constructed. This kind of convection is strictly defined as the Marangoni flow [33].

From the perspective of interface physicochemistry, the core mechanism of this phenomenon can be further explained, as the difference in surface tension at the interface is the source of shear stress. This shear stress, as a direct driving force, prompts the fluid to flow macroscopically in the direction of “low surface tension to high surface tension”, and its flow behavior follows the simplified form of the Navier–Stokes equation under interface conditions. Ultimately, a closed convective loop is formed under the influence of the gravitational field or boundary constraints, constituting a self-sustaining dynamic system [34]. By taking advantage of this convective effect mediated by the surface tension gradient, the Marangoni actuator can achieve autonomous movement in fluids without external mechanical drive. The essence of its movement mechanism is to achieve the directional transfer of momentum by regulating the interfacial tension gradient [35]. From the perspective of physical triggers, the triggering mechanisms of the Marangoni effect mainly include two types: temperature-gradient-driven and solute-diffusion-driven. Both achieve convection regulation by altering the spatial distribution of interfacial tension, as shown in Figure 1C(a,b).

The core thermodynamic basis of the Marangoni effect triggered by temperature gradients lies in the quantitative correlation between surface tension and temperature. When there is a temperature gradient

(1) ∇T≠0

on the surface of a liquid, the surface tension will form a gradient

(2) ∇σ≠0

accordingly. According to thermodynamic relations, the temperature coefficient of surface tension

(3) γ=dσ/dT

is negative, which means that an increase in temperature will lead to a decrease in surface tension, that is, the surface tension in the high-temperature region ($\sigma_{\mathrm{high}-T}$) is lower than that in the low-temperature region ($\sigma_{\mathrm{low}-T}$) [36]. This tension difference will generate shear stress

(4) τ=∂σ/∂x

along the gradient direction at the gas–liquid interface. This stress drives the surface fluid to flow from the low surface tension zone (high temperature) to the high surface tension zone (low temperature), while the bottom fluid flows in the opposite direction under the compensation of the pressure gradient, eventually forming a closed convection loop [29]. For instance, in a horizontally placed thin layer of liquid, if a local heat source is applied to form a linear temperature gradient

(5) ∇T=ΔT/L

then the interfacial tension distribution can be calculated through

(6) $$\sigma \left(T\right)=\sigma_{0}+\gamma (T-T_{0})$$

and then the flow velocity and flow field structure of the Marangoni flow can be predicted through fluid dynamics simulation. The flow velocity order usually reaches 10^−3^ to 10^−1^ m/s. It specifically depends on the intensity of the temperature gradient and the inherent properties of the liquid (such as viscosity and surface tension coefficient) [37].

The core of the Marangoni effect triggered by solute diffusion lies in the fact that the non-uniform distribution of solute molecules in the liquid alters the intermolecular forces at the interface, and the adsorption or dissolution of solute molecules directly regulates the magnitude of surface tension [38]. When a solute forms a concentration gradient

(7) ∇c≠0

on the liquid surface, a surface tension gradient

(8) ∇σ≠0

is generated accordingly. The relationship can be expressed as

(9) ∇σ=(dσ/dc)∇c

where dσ/dc is the surface tension concentration coefficient of the solute (for surfactants like SDS, dσ/dc is negative, that is, the surface tension in the high-concentration area is lower) [39]. This gradient-driven Marangoni flow is the main manifestation of this effect in multi-component systems and has important applications in fields like microscale mass transfer and interfacial reaction regulation [40]. For instance, in an aqueous solution containing SDS (sodium dodecyl sulfate), when SDS molecules form a concentration gradient on the droplet surface, the fluid in the high-concentration area (low surface tension) is driven to flow towards the low-concentration area (high surface tension), and the resulting vortex can significantly alter the deposition behavior of particles. Specifically, it is manifested as the suppression of the traditional “Coffee-ring effect”, as, through convection, the particles deposited at the edge are redispersed to the center of the droplet, eventually forming a uniform deposition spot. This phenomenon has been applied in technologies like high-precision printing and biochip preparation (Figure 1C(c)) [30].

In addition, the closed convection loop caused by solute diffusion (Figure 1C(d)) can precisely control the flow field intensity by regulating the matching relationship between the solute diffusion coefficient (D) and the interfacial tension gradient (∇σ), and its convection rate (v) satisfies (μ is the viscosity of the liquid), providing an efficient control method for active transport in microfluidic systems [41].

For thermal Marangoni actuators

(10) $$u∼(\Delta T\cdot \left|\gamma \right|\cdot L)/\mu$$

where ΔT is the temperature gradient,

(11) $$\left|\gamma \right|\approx 0.1\;mN/(m\cdot K)$$

for aqueous systems, L is the characteristic length, and

(12) μ≈1 mPa⋅s

yielding

(13) $$u∼10^{-2}\cdot \Delta T\cdot L$$

for typical

(14) ΔT∼10K

and

(15) L∼1 mm

For solutal driving,

(16) $$u∼(\Delta c\cdot \left|d\sigma /\mathrm{dc}\right|\cdot L)/\mu$$

with

(17) $$\left|d\sigma /\mathrm{dc}\right|\approx 1\;\mathrm{mN}\cdot m/\mathrm{kg}$$

are below CMC, leading to

(18) $$u∼10^{-2}\cdot \Delta c\cdot \mathrm{Lmm}/s$$

for

(19) $$\Delta c∼10\;\mathrm{kg}/m^{3}$$

This will provide intuitive design guidance for linking material/geometric parameters to performance.

Recent advances in mathematical modeling and computational simulations have enhanced the quantification and prediction of Marangoni actuator dynamics, bridging theoretical principles and experimental observations [42]. Careaga et al. developed a modeling framework for surfactant source-leak-induced Marangoni flow using thin film equations and lubrication theory. Via mixed finite element methods, they simulated spatiotemporal evolutions of liquid film height and surfactant concentrations, revealing flow pattern characteristics under source–leak interactions [43]. Carl et al. focused on thermocapillary driven rotation of micro-gears and the translation of asymmetric particles, constructing transient models coupling Navier–Stokes equations with heat transfer. They analyzed the impacts of gear tooth number, geometry, and size on motion speeds, identifying correlations between Reynolds number and rotational efficiency and effective translation of “Christmas tree” particles under illumination [44]. Potter et al. addressed temperature-dependent Marangoni-driven films, establishing simplified models incorporating temperature-dependent viscosity and surface tension via lubrication theory [45]. They uncovered unique flat steady-state solutions induced by temperature-dependent viscosity and clarified its influence on periodic and finite time blow-up solutions. These studies, using finite element and finite difference methods, provide critical theoretical and simulation support for understanding flow laws, stability, and performance optimization of Marangoni actuators.

**Figure 2:**
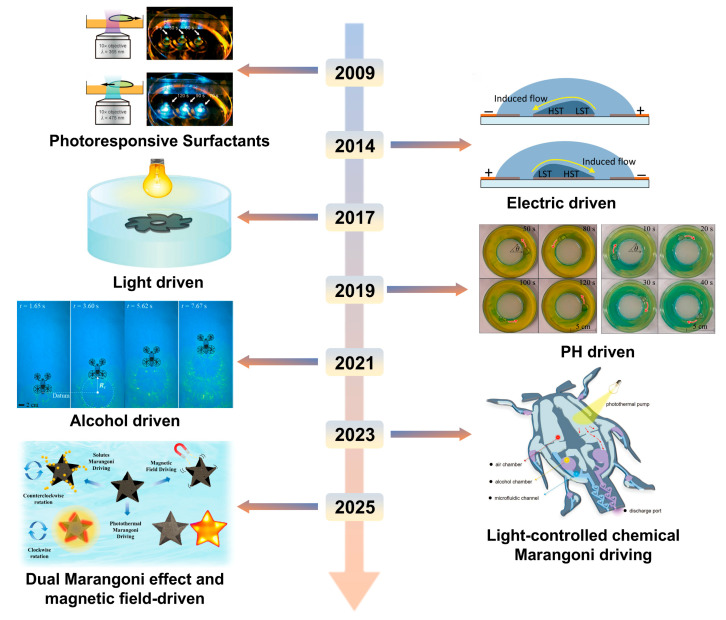
Temporal evolution and technological breakthroughs of the driving mechanism of the Marangoni actuator. Photoresponsive surfactant.

The advantages of Marangoni actuators are an important direction for future research (Figure 1D). Furthermore, the literature retrieved from 2009 to 2024 shows that a large number of Marangoni actuators with different propulsion mechanisms have emerged (Figure 2).

### 2.2. Material and Structural Design of Marangoni Actuators

Similar to the aforementioned mechanism, the core of the movement principle of the Marangoni actuator lies in the formation of a surface tension gradient with the surrounding fluid. In this process, the asymmetric structural design of the actuator is decisive for its functional realization. Specifically, temperature-gradient-based actuators construct surface tension gradients through the asymmetric distribution of external fields, while solute concentration gradient-based actuators achieve this via the asymmetric release of internal surfactants. This subsection will elaborate on the material selection and structural design of Marangoni actuators from the two dimensions of temperature gradient and solute diffusion.

#### 2.2.1. Design of Marangoni Actuator Based on Temperature Gradient

As mentioned, the motion of temperature-gradient-based Marangoni actuators stems from the photothermal Marangoni effect; utilizing the photoresponsiveness and excellent photothermal conversion ability of photothermal materials, local fluid temperature in the actuator contact area is elevated, inducing Marangoni flow to propel the actuator. Compared with the solute diffusion mechanism, photothermal Marangoni actuation based on photothermal materials features distinct characteristics like non-pollution and rapid response [46,47]. Moreover, compared with magnetic and acoustic heating, photothermal conversion materials enable efficient photothermal conversion and thermal response under simple conditions. Additionally, their strong programmability and wireless controllability have led to wide application in the motion control of Marangoni actuators in recent years [48].

**Figure 3:**
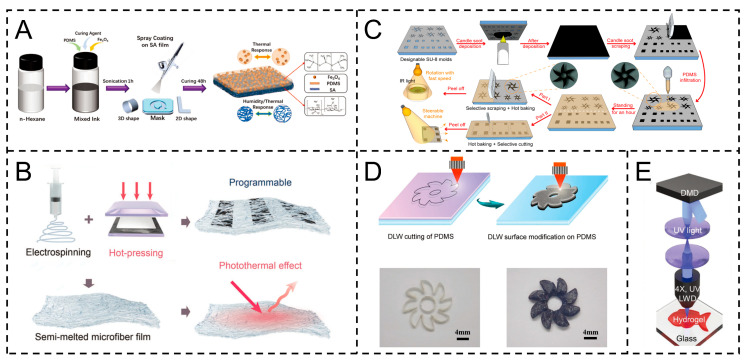
Preparation of Marangoni actuators based on temperature gradient. (**A**) The PDMS/Fe_3_O_4_@SA double-layer photothermal actuator was constructed through the mask-assisted spraying method. Reproduced from Reference [49] with permission from *Advanced Functional Materials*. (**B**) Preparation of black phosphorus (BP) enhanced programmable photothermal actuator through the electrospinning–hot-pressing method. Reproduced from Reference [12] with permission from *Advanced Materials*. (**C**) Preparation of candle ash (CS)/PDMS composite photothermal actuators through the soft lithography method. Reproduced from Reference [50] with permission from *Sensors and Actuators B: Chemical*. (**D**) Preparation of superhydrophobic PDMS photothermal actuators through direct laser writing (DLW). Reproduced from Reference [51] with permission from *Advanced Functional Materials*. (**E**) Preparation of CuS-doped PNIPAM hydrogel actuators with the assistance of digital micromirror devices (DMDs) and ultraviolet exposure. Reproduced from Reference [52] with permission from *Advanced Functional Materials*.

The heat generation mechanism of the photothermal Marangoni actuator is mainly the photothermal effect. Different from the actuator of solute diffusion, the photothermal Marangoni actuator is usually composed of sheet-like films. This sheet-like film is mainly made of photothermal conversion materials and substrate materials to reduce the weight of the actuator and enhance its photothermal conversion capability. For example, Sun et al. fabricated a bilayer actuator via mask-assisted spraying: PDMS/Fe_3_O_4_ composite (PIC layer) on sodium alginate (SA) films, with secondary curing. Fe_3_O_4_ enables efficient photothermal conversion, driving motion via the photothermal Marangoni effect (Figure 3A) [49]. Wu et al. fabricated the actuator via hot-pressing, using PVDF microfiber membranes (MFF), black phosphorus (BP), and copper–nickel conductive fabrics. BP enhances photothermal conversion, inducing motion via the photothermal Marangoni effect (Figure 3B) [12].

Similarly, Wang et al. fabricated the actuator via soft lithography, combining candle ash (CS) with PDMS. CS, an efficient photothermal material, enables photothermal conversion, driving motion via the photothermal Marangoni effect (Figure 3C) [50]. Moreover, Wang et al. fabricated superhydrophobic actuators via DLW on PDMS. Carbonized PDMS layers enable photothermal conversion, driving motion via the photothermal Marangoni effect (Figure 3D) [51]. Pan et al. fabricated the LTMA (Light-driven transparent Marangoni actuator) via UV exposure, using PNIPAM (Poly(N-isopropylacrylamide))hydrogels doped with CuS nanoparticles and combined with DMD (digital micromirror device). CuS enables efficient photothermal conversion, inducing movement via the photothermal Marangoni effect (Figure 3E) [52].

#### 2.2.2. Design of Marangoni Actuator Based on Solute Concentration

Based on the release capacity of solutes in the fluid, the local fluid solute concentration in the contact area of the actuator is increased, causing a change in surface tension and thereby inducing the generation of Marangoni flow, ultimately achieving the propulsion of the Marangoni actuator based on the solute concentration gradient. Typically, the asymmetric release of surfactants carried by the actuator in the fluid causes a surface tension gradient, and the Marangoni actuator based on the solute gradient achieves motion [53].

**Figure 4:**
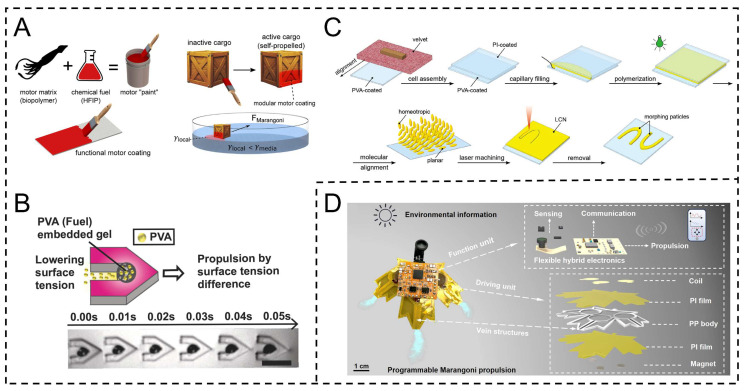
Preparation of Marangoni actuator based on solute concentration gradient. (**A**) Solute controllable release Marangoni actuator based on SRT protein matrix. Reproduced from Reference [54] with permission from *ACS Applied Materials & Interfaces*. (**B**) PVA fuel-mediated Marangoni actuator. Reproduced from Reference [55] with permission from *Nature Communications*. (**C**) Programmable solute release Marangoni actuator regulated by light-controlled liquid crystal elastomer (LCN). Reproduced from Reference [56] with permission from *Advanced Functional Materials*. (**D**) Integrated Marangoni actuator for electrically controlled solute release. Reproduced from Reference [61] with permission from *eScience*.

Therefore, the selection of surfactants plays a crucial role in ensuring the smooth movement of actuators. Lin et al. developed a solute-gradient Marangoni actuator with hexafluoroisopropanol (HFIP) as the preferred solute. HFIP, with a high linearized surface tension parameter, forms a synergistic effect with the SRT protein matrix, via which it is controllably released. SRT’s dynamic nanostructural changes enable solute self-regulation, inducing surface tension gradients for actuation (Figure 4A) [54]. Choi et al. developed a light-patterned Marangoni swimmer using polyvinyl alcohol (PVA) as fuel (reducing surface tension when dissolved) and hydrophobic polyurethane acrylate as the main body. PVA, loaded in PEGDA hydrogel, is controllably released to induce surface tension gradients for propulsion; PVA’s biodegradability aids application (Figure 4B) [55]. Huang et al. developed a solute-gradient Marangoni actuator with HFIP as fuel and SRT protein as the matrix. Photochemical LCN (liquid crystal elastomer), as a shape-deforming structure, regulates fuel release via light, enabling programmable motion modes (Figure 4C) [56].

Although the self-release mechanism of the solute provides the fundamental driving force for the movement of the Marangoni actuator, its inherent dynamic instability has gradually become the key bottleneck restricting the performance improvement of the actuator [57]. During the self-release process, the release kinetics of the solute is highly dependent on the random fluctuations of environmental parameters (such as local fluid viscosity, temperature field distribution, and the solute’s own diffusion coefficient), resulting in the concentration gradient of the surfactant in the fluid showing non-steady-state spatiotemporal evolution characteristics [58]. Not only is it difficult to maintain a constant release rate, but the peak position and intensity of the concentration gradient will also drift unpredictably over time, which in turn causes intermittent disturbances of the Marangoni flow and random shifts in the actuator’s motion trajectory, seriously reducing the accuracy and repeatability of motion control. To break through this limitation, the electrically controlled solute release strategy emerged [59]. By actively modulating the solute release process with an external electric field, the release rate can be digitally and precisely regulated and dynamically responded to in real time [60]. This not only effectively suppresses the interference of environmental disturbances in the stability of the concentration gradient but also endows the solute release process with higher spatiotemporal resolution by adjusting the electric field intensity and action timing. It provides core technical support for the construction of high-performance and highly controllable Marangoni actuators. For instance, Zhou et al.’s S-aquabots actuator employs double-layer polyimide (PI) films, 3D-printed foamed polypropylene (PP) frames, copper coils (on PI), NdFeB (N52) magnets, flexible printed circuit boards (FPCBS), and silicone (for bonding). Coil-energized magnetic fields deform PI films to release ethanol via channels; power cutoff sucks back ethanol. These materials enable precise digital motion control (Figure 4D) [61].

These representative photothermal Marangoni actuators, characterized by distinct material combinations (e.g., candle ash/PDMS, carbonized PDMS, CuS-doped PNIPAM hydrogels) and photothermal conversion-based trigger mechanisms, are systematically summarized in Table 1, which outlines their core material components and trigger modes for clear comparison.

#### 2.2.3. Hydrogel/Organogel-Based Marangoni Actuators

Hydrogels have emerged as pivotal materials in the development of Marangoni actuators, capitalizing on their inherent stimuli responsiveness, tunable network architectures, and dynamic interfacial behaviors to enable versatile self-propulsion at liquid–air interfaces. These systems primarily rely on either solute concentration gradients (via controlled release of surfactants) or temperature gradients (via photothermal conversion) to induce interfacial tension gradients, thereby driving Marangoni flow and actuating locomotion [62].

A defining advantage of hydrogels lies in their unique controllable swelling–deswelling kinetics and porous network structures, which facilitate the encapsulation of functional solutes (e.g., surfactants or chemical fuels) and enable precise spatiotemporal controlled release—a critical prerequisite for establishing the solute concentration gradients that underpin Marangoni-driven motion [63]. For instance, the hydrogel system developed by Zhou et al. is constructed through synergistic interactions between MXene, chitosan, and vanillin via Schiff base bonds and hydrogen bonds. In this design, vanillin, as a natural surfactant, achieves regulated release through the cleavage of dynamic bonds, thereby constructing stable concentration gradients within the system. The three-dimensional porous architecture of the hydrogel further promotes efficient diffusion of vanillin, providing a sustained driving force for Marangoni propulsion [19]. Additionally, the buoyancy of the gel is precisely modulated through density engineering, while its nanoconfined channels optimize ion transport efficiency, offering robust support for aqueous auxiliary power generation processes.

Furthermore, the tunable stimulus responsiveness of hydrogels to external cues (e.g., light, temperature, pH) enables synergistic coupling with the Marangoni effect, laying the foundation for precise regulation of actuation behaviors [64]. Zheng et al. prepared PNIPAM-based hydrogels by covalently incorporating oxazine derivatives (OX-1 and OX-2) into the polymer network via free radical copolymerization. Under visible light irradiation (450 nm or 520 nm), the oxazine moieties exhibit efficient photothermal conversion capabilities, generating localized temperature gradients that reduce the interfacial tension of the irradiated region. This mechanism triggers unidirectional movement, turning, and rotational motion, with stable performance maintained over 10 heating–cooling cycles [65].

For gel-specific design, key parameters include crosslink density, which regulates swelling kinetics to control solute release rates and gradient stability; porosity, which augments fuel encapsulation capacity and mass transport efficiency; density/buoyancy modulation via material engineering to adapt to diverse fluid environments; and surface wettability, which optimizes interfacial interactions with the fluid medium to ensure efficient transduction of tension gradients into directional locomotion. Collectively, these integrated properties establish hydrogels as an ideal material platform for advancing Marangoni actuation across multiple length scales. Table 2 summarizes the core performance parameters of different gel-based Marangoni actuators for intuitive comparison.

## 3. The Driving Method of the Marangoni Actuator

Consistent with the aforementioned core principle, Marangoni actuators essentially drive via surface tension gradients constructed through external fields or internal forces. The characteristic differences of driving modes crucially impact gradient formation efficiency and regulation accuracy. Specifically, light-driven methods achieve non-contact gradient construction via local characteristic transformations of photoresponsive materials (such as photoisomerization and photothermal effects); chemical-driven methods form gradients based on asymmetric diffusion and reaction kinetics of chemicals; and electro-driven methods dynamically regulate gradients by controlling interface charge distribution via electric fields. This section elaborates on these three methods regarding gradient mechanisms, performance, and implementation paths.

### 3.1. Light-Driven

Light-driven Marangoni actuators are a type of intelligent micro-nano device that achieves autonomous motion by using light-controlled interfacial tension gradients. Their core mechanism is based on the synergistic effect of the Marangoni effect and light-responsive materials. When light of a specific wavelength is irradiated on the surface of the actuator or the surrounding medium, the light energy is absorbed by light-sensitive substances (such as azobenzene derivatives, carbon-based nanomaterials, etc.) The surface tension of the local area is changed through processes like photoisomerization, photothermal conversion, or photochemical decomposition [66].

**Figure 5:**
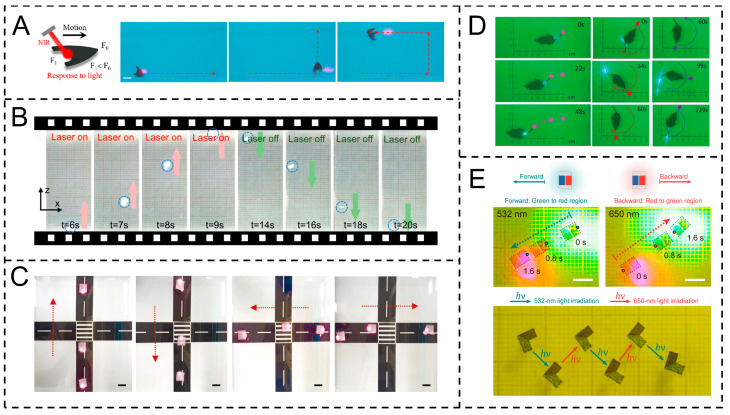
Light-driven Marangoni actuator. (**A**) Photothermal drive of graphene/PDMS composite actuators. Reproduced from Reference [68] with permission from *ACS Nano*. (**B**) Photothermal driving of CuS-nanoparticle-doped PNIPAM hydrogel actuators. Reproduced from Reference [52] with permission from *Advanced Functional Materials*. (**C**) Photothermal drive of black phosphorus (BP)-doped PVDF microfiber membrane actuators. Reproduced from Reference [12] with permission from *Advanced Materials*. (**D**) Photothermal drive of PI tape laser-induced graphene (LIG) patterned actuators. Reproduced from Reference [69] with permission from *Advanced Functional Materials*. (**E**) Photothermal drive of BOPP substrate PDMS-doped OR/PB (dual-wavelength response) actuators. Reproduced from Reference [70] with permission from *Science Advances*.

For example, Wang et al. fabricated a light-driven Marangoni actuator using graphene/PDMS composites. Graphene’s photothermal conversion raises local water temperature under infrared laser, reducing surface tension to form gradients, driving motion; a dual-pointed tail enables directional control (Figure 5A) [68]. Pan et al.’s transparent LTMA, a light-driven Marangoni actuator, uses PNIPAM hydrogel with CuS nanoparticles. Near-infrared laser irradiation enables CuS to convert light to heat, inducing surface tension gradients for programmable motion, with transparency from ultra-thin structures and low nanoparticle concentration, and porous hydrogel enabling molecule adsorption for signal enhancement while PNIPAM’s softness enhances biocompatible comfort (Figure 5B) [52]. Similarly, Wu et al.’s light-driven Marangoni actuator uses BP-doped PVDF microfiber films with conductive fabrics. Infrared laser irradiation enables BP to convert light to heat, inducing surface tension gradients for controllable water surface motion (Figure 5C) [12]. Wang et al.’s light-driven Marangoni actuator uses LIG (laser-induced graphene) patterns on PI tape. Light (laser/sunlight) enables LIG’s photothermal conversion, inducing surface tension gradients; patterned LIG enables directional motion (Figure 5D) [69].

Despite the significant advancements exemplified above, light-driven Marangoni actuators still face inherent limitations that constrain their practical deployment in complex scenarios. A primary challenge lies in the constrained light penetration depth in turbid or biologically complex media (e.g., tissue environments or high-concentration solutions), which weakens the efficiency of photothermal conversion or photoisomerization processes and restricts the effective working range of the actuator. Additionally, maintaining stable surface tension gradients typically requires continuous illumination, as the cessation of light input leads to rapid dissipation of temperature or photochemical gradients, limiting the autonomy of motion control. To address these limitations, emerging strategies have been developed, including multi-wavelength responsive materials, such as the OR/PB (Organic dye/phosphorus-based material)-doped PDMS in Hou et al.’s design, which enables adaptive adjustment to different light penetration depths by responding to distinct wavelengths (532 nm and 650 nm), expanding operational versatility. Meanwhile, upconversion nanoparticles, which can convert low-energy near-infrared light (with deeper tissue penetration) into high-energy visible light, have shown potential in enhancing light utilization efficiency in opaque environments, providing a promising pathway to overcome penetration depth barriers (Figure 5E) [70].

### 3.2. Chemically Driven

Chemically driven Marangoni actuators are a type of intelligent micro-nano device that achieves autonomous motion by chemically regulating the interfacial tension gradient. Their core mechanism is based on the synergistic effect of the Marangoni effect and changes in the chemical environment. Here, we will introduce from three chemical substances: alcohol, pH, and sodium dodecyl sulfate (SDS). When alcohol concentration gradients, pH value fluctuations, or distribution differences of surfactants, such as sodium dodecyl sulfate (SDS), act on the surface of the driver or the surrounding medium, chemically sensitive components (such as pH-responsive polymers, amphiphilic molecular modification materials, etc.) will shift through selective adsorption, ionization equilibrium, or intermolecular interactions, triggering a change in surface tension in a local area [71].

#### 3.2.1. Alcohol-Driven

Alcohol is chosen as the driving substance for the chemically driven Marangoni actuator mainly due to its unique physicochemical properties. Alcohol (such as ethanol) has good miscibility with water, but its surface tension is significantly lower than that of water. When alcohol forms a local concentration gradient in the medium, it will rapidly cause an asymmetric distribution of interfacial tension, thereby efficiently driving the flow of Marangoni [72]. Meanwhile, the volatility and diffusion rate of alcohol are easy to regulate. By controlling its release amount or diffusion path, the movement speed and direction of the actuator can be precisely regulated [73].

**Figure 6:**
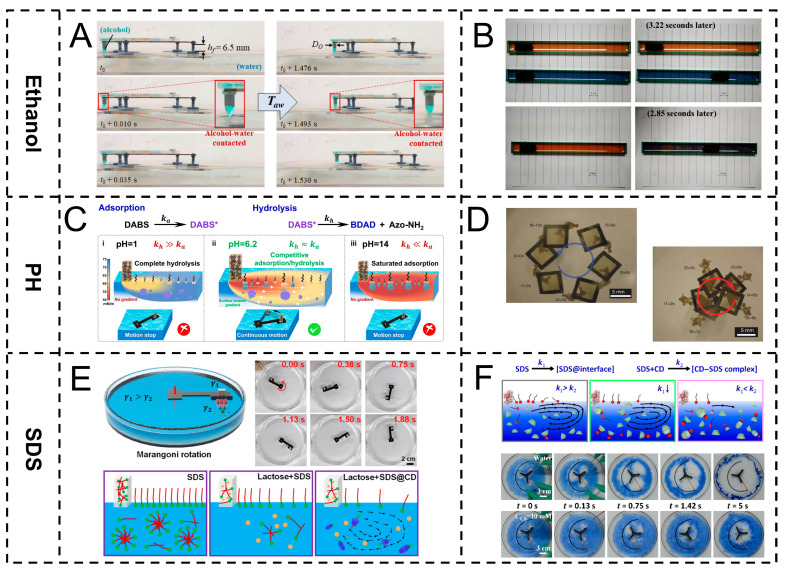
Chemically driven Marangoni actuator. (**A**,**B**) Alcohol-driven. (**A**) Controllable alcohol gradient drive. Reproduced from Reference [74] with permission from *Scientific Reports*. (**B**) Ph-responsive ethanol drive. Reproduced from Reference [75] with permission from Small. (**C**,**D**) pH-driven. (**C**) Regulation of competitive adsorption/hydrolysis of DABS molecules. Reproduced from Reference [81] with permission from *Angewandte Chemie International Edition*. (**D**) Chiral molecular crystal pH dissociation drive. Reproduced from Reference [82] with permission from *Nature Communications*. (**E**,**F**) pH drive. (**E**) SDS gradient-driven multi-engine rotor. Reproduced from Reference [87] with permission from *ACS Applied Materials & Interfaces*. (**F**) β-cyclodextrin regulates SDS drive. Reproduced from Reference [88] with permission from *CCS Chemistry*.

Kwak et al. developed a Marangoni-driven surface robot using alcohol (such as 3-methyl-1-butanol) as the driving substance, leveraging its surface tension difference with water. A self-priming microfluidic pump releases alcohol (as droplets or columnar structures) to create surface tension gradients, enabling autonomous movement via the Marangoni effect. Alcohol release rate and diffusion, regulated by porous media, nozzle diameter, and foot pad height, affect motion performance (Figure 6A) [74]. In addition, the alcohol-induced Marangoni effect holds significant importance in microfluidic research. Its core value lies in providing contactless, low-energy consumption and a precisely controllable driving mechanism for microscale fluid manipulation. For instance, Xiao et al.’s device realizes microfluidic driving via the alcohol-triggered Marangoni effect, using 90% ethanol as a low-surface-energy driver stored in reservoirs. pH-responsive nickel mesh controls ethanol release (blocked in acid, enabled in alkali); alcohol–water surface tension differences form gradients to drive superhydrophobic hulls, with higher ethanol concentrations enhancing driving (Figure 6B) [75].

#### 3.2.2. pH-Driven

The core mechanism of pH value as a Marangoni actuator lies in its precise regulation of the dissociation state of interfacial active substances and interfacial adsorption behavior [76]. The hydrophilic–hydrophobic equilibrium state of surface-active molecules containing ionizable groups (such as carboxyl and amino groups) changes significantly with pH values [77]. Under acidic conditions, protonation enhances the hydrophobicity of molecules, promoting their adsorption towards the interface and reducing the interfacial tension. However, in an alkaline environment, deprotonation leads to an increase in the hydrophilicity of molecules, weakening the interfacial adsorption capacity and raising the interfacial tension [78]. This pH-dependent interfacial tension response characteristic enables local pH gradients to be directly converted into interfacial tension gradients, thereby driving the Marangoni flow [79]. In addition, pH regulation is reversible, non-invasive, and easy to operate. By regulating the acid–base distribution, the flow behavior can be dynamically modulated, providing an efficient and controllable actuation mechanism for microscale fluid drive [80].

Zhu et al. regulated a Marangoni actuator driving via pH control over the competitive interfacial adsorption and hydrolysis of the superamphiphilic molecule DABS. DABS, with hydrophilic–hydrophobic moieties linked by pH-responsive Schiff bases, exhibits interfacial activity only in an intact form, while hydrolysis products are inactive. Neutral pH (≈6.2) balances the two rates (k_a_ ≈ k_h_), maintaining stable surface tension gradients for actuation (ON). Acidic (pH = 1, k_h_ ≫ k_a_) or alkaline (pH = 14, k_a_ ≫ k_h_) conditions disrupt the balance, eliminating gradients and halting movement (OFF) (Figure 6C) [81]. Similarly, Carmeli et al. drove the unidirectional rotation of underwater micro-motors (chimots) via pH-regulated dissociation of chiral molecular crystals (sinkonin/sinkonidin). Acidic/neutral conditions (pH ≤ 7) induce the protonation of crystal nitrogen sites, enhancing coulomb repulsion and water affinity to promote asymmetric molecular release, forming gradients that drive rotation via the Marangoni effect. Highly alkaline conditions (pH = 10) inhibit dissociation, halting rotation (Figure 6D) [82].

#### 3.2.3. SDS-Driven

The core mechanism of sodium dodecyl sulfate (SDS) as the solvent driven by the Marangoni actuator lies in the highly efficient interfacial activity endowed by its amphiphilic molecular structure and the dynamic regulation ability of the solvent surface tension gradient [83]. The hydrophobic dodecyl chain and hydrophilic sulfate group (-OSO_3_^−^ Na^+^) of SDS can be directionally adsorbed at the gas–liquid interface, significantly reducing the surface tension of the solvent through a densely arranged molecular film (for example, reducing the surface tension of pure water from 72 mN/m to 30–40 mN/m) [84]. SDS interface concentration differences induce heterogeneous adsorption, forming surface tension gradients (low in high-concentration areas, high in low-concentration areas). The Marangoni effect drives solvent flow along gradients to balance interfacial energy [85]. In addition, the concentration-dependent adsorption behavior of SDS below the critical micelle concentration (CMC) can be dynamically maintained by regulating the release rate to achieve a gradient, while the micellar effect above CMC avoids rapid interface saturation, further ensuring the sustainability of the drive and making it an effective actuating unit for controllable solvent drive [86].

Lu et al. fabricated a controllable multi-engine Marangoni rotor for small generators, with SDS as the driver. SDS, via its amphiphilic structure (hydrophobic dodecyl chain and hydrophilic sulfonic acid group), adsorbs directionally at the gas–liquid interface to form a dense molecular film, reducing local surface tension. SDS release from the actuator’s fuel end creates concentration differences, leading to heterogeneous adsorption and surface tension gradients (low in high-concentration areas, high in low-concentration areas), which drive fluid flow via the Marangoni effect to propel the actuator (Figure 6E) [87]. Similarly, Cheng et al. employed SDS as fuel for Marangoni actuators; its amphiphilic structure enables directional adsorption at gas–liquid interfaces, forming molecular films to reduce local surface tension. SDS release creates concentration differences, generating surface tension gradients that drive motion via the Marangoni effect. Introducing β-cyclodextrin removes excess interfacial SDS, extending actuation life from 55 s to 2400 s (Figure 6F) [88].

### 3.3. Electrically Driven

Electrically driven Marangoni actuators are a type of precision micro-nano device that achieves controllable motion by regulating the interfacial tension gradient with electrical signals. Their core mechanism is based on the coupling effect of the Marangoni effect and the electrical response system [89]. When a specific intensity of electrical signal acts on the surface of the actuator or the surrounding electrolyte, electrical energy promotes the distribution reconstruction or property transformation of the surface-active substances (such as ionic surfactants, conductive polymers, etc.) at the interface between the liquid metal and the electrolyte through electrochemical reactions, ion migration, electric bilayer regulation, etc., thereby changing the surface tension in the local area. This further triggers the Marangoni effect (directional flow or movement driven by the gradient of surface tension) [90].

**Figure 7:**
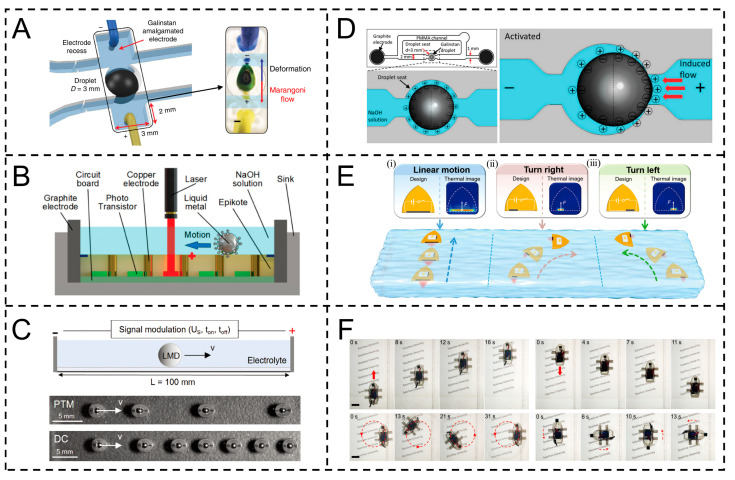
Electrically driven Marangoni actuator. (**A**) Droplet movement regulated by electric bilayer (EDL). Reproduced from Reference [91] with permission from *Nature Communications*. (**B**) Potential gradient drive of optical–electrical coupling. Reproduced from Reference [92] with permission from *Materials Horizons*. (**C**) Behavior of droplet/liquid metal marbles regulated by electric field. Reproduced from Reference [93] with permission from *Advanced Functional Materials*. (**D**) Liquid flow control driven by alternating electric fields. Reproduced from Reference [94] with permission from *Proceedings of the National Academy of Sciences*. (**E**) Driving of the electroinduced Joule heat effect. Reproduced from Reference [95] with permission from Small. (**F**) Continuous electrowetting (CEW) liquid metal engine. Reproduced from Reference [96] with permission from *Advanced Materials Technologies*.

For instance, Zavabeti et al. achieved droplet driving via the Marangoni effect by altering liquid metal–electrolyte interface electric bilayer (EDL) properties and regulating surface tension gradients using the Lippmann equation. Low ion gradients induce droplet deformation (ratio up to 1.46), and high gradients drive directional Marangoni flow (with a maximum flow rate of 1.74 mm/s), with a pH threshold (about 13). External voltage and salt enhance EDL, enabling 25 mm/s self-propulsion (Figure 7A) [91]. Ren et al. used an infrared laser to activate a phototransistor, generating an electric potential gradient toward the copper electrode, altering the electric bilayer charge distribution at the liquid metal–electrolyte interface. Via the Young–Lippmann equation, this forms a surface tension gradient, producing Marangoni forces to drive droplets (up to 55 mm/s), enabling precise trajectory tracking and multi-droplet control (Figure 7B) [92]. Based on the Lippmann-equation-derived electric capillary effect, Fuchs et al. regulated the droplet–electrolyte interface double electric layer (EDL) via an electric field, inducing surface tension gradients to generate Marangoni flow for directional motion. Pulse time modulation (PTM) optimized motion by tuning voltage conduction (propelling droplets) and turn-off (dissolving surface oxides) times, while direct current (DC) caused rapid deceleration due to persistent oxide accumulation (Figure 7C) [93]. Tang et al. altered Galinstan liquid metal–electrolyte interface electric bilayer (EDL) properties via alternating electric fields, regulating surface tension gradients using the Lippmann equation. Higher downstream hemisphere tension creates pressure differences, driving liquid via the Marangoni effect. Furthermore, 200 Hz square waves prevent oxide accumulation, enabling 5400 μL/min flow (Figure 7D) [94].

In macro scenarios, the driving mechanism of this type of actuator can be further expanded. By means of electric heating, the Joule effect is utilized to raise the temperature of the local area, altering the surface tension distribution at the interface between the liquid metal and the medium, forming a gradient field and driving the Marangoni flow. For instance, Lin et al. employed the Joule heat effect of superaligned carbon nanotubes (SACNT) to rapidly elevate local actuator temperatures, creating temperature gradients that alter water surface tension distributions, generating gradients to drive linear, turning, and bidirectional rotational motions. Regulating SACNT position and electric heating power enables motion control, enhancing stability and anti-interference and simplifying design for complex tasks (Figure 7E) [95].

Meanwhile, the drive mode of the liquid metal engine based on electrical control directly regulates the morphological changes and interfacial tension gradients of the liquid metal through an electric field and combines the Marangoni effect to achieve power output, thereby realizing efficient mechanical motion transmission and energy conversion at the macroscopic scale. Li et al. applied voltage to gallium-based liquid metals (such as Galinstan), using the continuous electrowetting (CEW) effect to directly regulate their morphology and induce interfacial tension gradients, triggering Marangoni flow to drive electrolyte flow. Channel-constrained backward water jets generate thrust, adjustable via droplet size, electrode gap, etc. Voltage polarity/PWM duty cycle enable complex motions, with no mechanical parts (Figure 7F) [96].

Regarding response speed light-driven systems, such as photothermal Marangoni actuators integrated with CuS or BP, they typically exhibit sub-second to second-level response owing to rapid photothermal conversion, whereas chemical-driven modes tend to show slower kinetics constrained by solute diffusion rates ranging from tens of seconds to minutes, and electrically driven actuators achieve millisecond to second-level response through electric field modulation. In terms of controllability, light-driven methods offer superior spatiotemporal resolution via laser positioning, and power tuning electrically driven systems enables digital and real-time regulation through voltage or pulse modulation. In contrast, chemical-driven actuators face challenges in maintaining stability due to fuel depletion and environmental fluctuations, though such issues have been mitigated through supramolecular strategies, such as β-cyclodextrin-regulated SDS release. To systematically quantify and cross-compare these performance nuances across representative architectures, Table 3 consolidates key metrics of Marangoni actuators spanning light-driven, chemical-driven, and other paradigms, enabling direct evaluation of their operational boundaries and technological trade-offs.

For environmental adaptability, light-driven actuators excel in non-invasive scenarios like biomedical microenvironments, yet they may be limited by light penetration depth. Chemical-driven systems are suitable for autonomous operation, but they require compatibility with chemical fuels. Electrically driven actuators provide precise control but depend on electrolyte environments and may introduce electrochemical interference. This integrated comparison consolidates scattered insights into a coherent framework, highlighting the complementary characteristics of each driving mode and offering strategic guidance for material selection and application-specific design.

## 4. The Applications of Marangoni Actuators

### 4.1. Applications at the Microscopic Scale

The Marangoni actuator has demonstrated extensive application value in the microscopic field due to its micro-scale fluid motion characteristics induced by surface tension gradients. In terms of microscopic salt ion transport and anti-crystallization, it can drive the directional migration of salt ions by regulating the interfacial tension gradient, reduce the formation of local high-concentration areas, and thereby inhibit the occurrence of crystallization phenomena.

**Figure 8:**
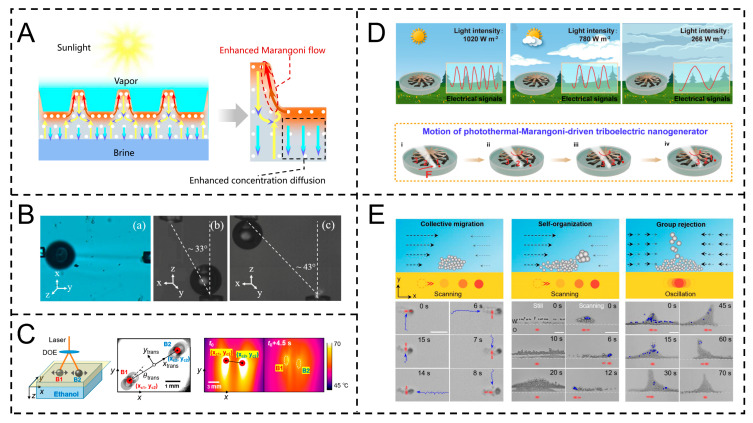
Microscale application of Marangoni actuators. (**A**) Regulation of salt ion transport in solar seawater desalination. Reproduced from Reference [97] with permission from *Science Advances*. (**B**) Precise spatial driving of microbubbles. Reproduced from Reference [98] with permission from *Optics Express*. (**C**) Autonomous movement and trajectory regulation of microbubbles. Reproduced from Reference [99] with permission from *Proceedings of the National Academy of Science*. (**D**) Microscopic energy conversion and light intensity sensing. Reproduced from Reference [100] with permission from *ACS Applied Materials & Interfaces*. (**E**) Behavioral regulation of biocompatible microcommunities. Reproduced from Reference [101] with permission from *Laser & Photonics Reviews*.

For instance, Wang et al. exploited the synergistic effect of solutes and the thermal Marangoni effect. By designing the side walls of a waffle evaporator (WSE), they utilized the concentration gradient (solute Marangoni effect) and temperature gradient (thermal Marangoni effect) generated by evaporation to trigger synergistic convection, accelerating the migration of salt ions from the evaporation surface to the bulk solution. This avoids salt crystallization from blocking the evaporator at the microscopic scale to achieve efficient solar seawater desalination (Figure 8A) [97]. For the precise driving and migration of microscopic bubbles, it can utilize the spatial differences in surface tension to precisely control the direction and speed of bubble movement, meeting the demand for precise regulation of bubble position at the microscale. Ortega-Mendoza et al. heated silver nanoparticles at the ends of optical fibers with a laser to generate a local temperature gradient in ethanol, triggering a surface tension gradient and forming a Marangoni force (up to 400 nN) to drive microbubbles (radius 110 μm) to migrate in two-dimensional/three-dimensional space (at a speed of 238 mm/s). The directional manipulation of bubbles in the microfluidic system has been achieved (Figure 8B) [98].

In the autonomous movement and trajectory regulation of microscopic bubbles, by taking advantage of the dynamic changes in surface tension caused by environmental factors (such as temperature, concentration, etc.), the bubbles can generate autonomous movement, and the movement trajectory can be effectively guided through preset interface conditions. Hu et al. irradiated a liquid with a laser to generate a temperature gradient. Through the thermal Marangoni effect, bubbles spontaneously oscillated horizontally at the solid–liquid interface (at a frequency of 24 Hz), synchronizing with the interface temperature and fluid flow. Meanwhile, the symmetry breaking of the vortex pairs causes the bubble trajectories to rotate. This effect can be applied to the autonomous motion design of micro-robots, the directional delivery of drug carriers, etc., to achieve contactless micro-motion regulation (Figure 8C) [99]. In the field of microscopic energy conversion and signal sensing, Marangoni actuators can convert the energy of surface tension gradients into other forms of energy, and, at the same time, they can sense external signals by monitoring the changes in fluid motion states, achieving the dual functions of energy conversion and signal detection. Liu et al. utilized black silicon to convert light energy into thermal energy, and, through the photothermal Marangoni effect, they triggered fluid flow, driving the rotor (equipped with a polytetrafluoroethylene friction layer) to rotate, achieving the conversion of light, mechanics, and electrical signals (with a peak voltage of 2.35 V). This effect makes the electrical signal period of the generator linearly related to the light intensity, achieving microscopic sensing scenarios, such as sunlight intensity monitoring (Figure 8D) [100].

In terms of biocompatible micro-group behavior regulation, based on biofriendly materials and driving mechanisms, it can regulate the movement behavior of biological micro-scale groups (such as cells, microorganisms, etc.), guiding them to move or gather in specific patterns, providing support for micro-manipulation in fields like biomedicine. Shi et al. induced reverse Marangoni flow (cold flow) at the water/silicone oil interface through a laser-induced temperature gradient, driving micro-particles (200 nm to 2 μm) or bacteria to form microcommunities, achieving intelligent behaviors, such as collective migration, size self-organization, and group rejection. This effect causes no thermal damage and enables biomedical microscopic applications in targeted drug delivery (such as single-cell delivery of sirNA-loaded particles) (Figure 8E) [101].

### 4.2. Applications at the Macroscopic Scale

The Marangoni actuator, relying on the dynamic regulation mechanism of the interfacial tension gradient, has demonstrated cross-disciplinary application potential at the macroscopic scale. In essence, these actuators precisely manipulate interfacial energy distribution to enable innovations in both material assembly and functional operation. It widely covers key fields, such as macroscopic supramolecular assembly, cargo transportation, programmable motion control, energy conversion, targeted drug delivery, environmental cleaning, and sensor detection, providing a novel approach to solve dynamic regulation challenges in complex systems.

#### 4.2.1. Macroscopic Supramolecular Assembly

In the field of macroscopic supramolecular assembly (MSA), it can guide the dispersed phase particles to achieve ordered aggregation and structural reconstruction by dynamically regulating the tension distribution at the liquid–liquid or liquid–gas interface, providing a precisely controllable assembly path for constructing macroscopic functional materials with complex topological structures.

**Figure 9:**
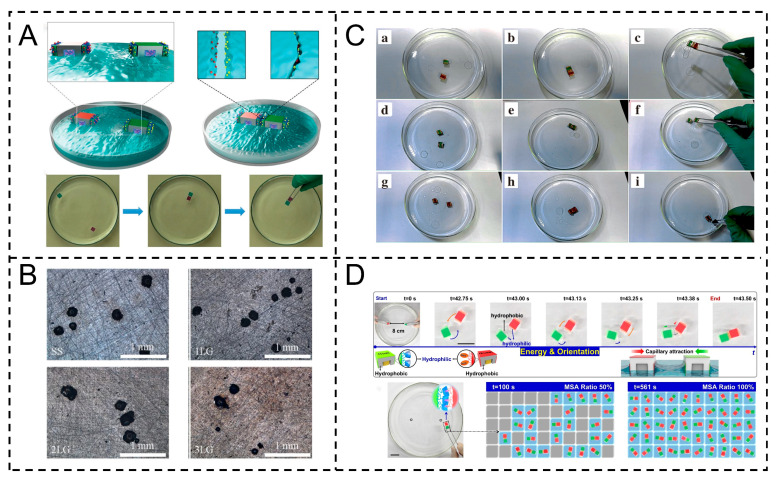
Macroscopic supramolecular assembly strategies and applications mediated by the Marangoni effect. (**A**) Precise and misaligned assembly of subject–object identification and regulation. Reproduced from Reference [102] with permission from *Angewandte Chemie International Edition*. (**B**) Self-assembly of graphene nanocoatings with different numbers of layers. Reproduced from Reference [103] with permission from *Chinese Chemical Letters*. (**C**) Density-regulated spontaneous motion assembly system. Reproduced from Reference [104] with permission from *Angewandte Chemie International Edition*. (**D**) pH-responsive hydrolysable fuel-driven assembly. Reproduced from Reference [105] with permission from *Angewandte Chemie International Edition*.

For instance, Cheng et al. used CD-SDS host–guest recognition to regulate surfactant adsorption, extending Marangoni motion lifetime and enhancing building block energy. Their building block design achieves 100% misalignment-free assembly, enabling large-scale structured material preparation (Figure 9A) [102]. Ye et al. fabricated layer-by-layer graphene nanocoatings via Marangoni effect self-assembly, and 0.1 mg/mL of graphene/alcohol dispersion, ultrasonically treated to avoid aggregation, was added to water. The surface tension gradient drove alcohol and graphene to spread; hydrophobic sheets self-assembled into uniform single-layer films, fished onto pretreated stainless steel, dried at 60 °C, and repeated for multilayers (Figure 9B) [103].

Similarly, Xiao et al. achieved spontaneous movement of PDMS building blocks via the Marangoni effect, driving them to interaction distances at air–water or water–perfluoronaphane interfaces, avoiding the randomness of traditional external-energy-driven methods. Adjusting block density (1.1–1.9 g/cm^3^) alters interface curvature, transforming assembly forces for precise control of linear/L-shaped ordered structures. Cyclodextrin–azobenzene recognition stabilizes assemblies, enhancing anti-shaking stability and enabling ordered bulk supramolecular material preparation (Figure 9C) [104]. In addition, Lu et al. synthesized hydrolyzable superamphiphilic SBBS as Marangoni self-propelled fuel, with a Schiff base structure enabling rapid interfacial adsorption (to generate tension gradients) and timely hydrolysis (to avoid over-aggregation), extending building block movement life to over 1100 s (vs. <100 s for SDS). MSA building blocks (SBBS, host–guest-modified PDMS, EPS cubes) form detachable ordered dimers via capillary alignment and host–guest recognition; pH = 2 enhances assembly efficiency by 24%, facilitating ordered millimeter-scale functional material preparation (Figure 9D) [105].

#### 4.2.2. Cargo Transportation

In terms of goods transportation, by taking advantage of the directional movement characteristics driven by the interfacial tension difference, this driver can achieve non-contact transportation of macro-scale loads (such as micro-nano devices, biological samples, etc.), and the movement trajectory can be regulated in real time through external stimuli (such as temperature and light), significantly enhancing the flexibility of micro-operations and logistics systems.

**Figure 10:**
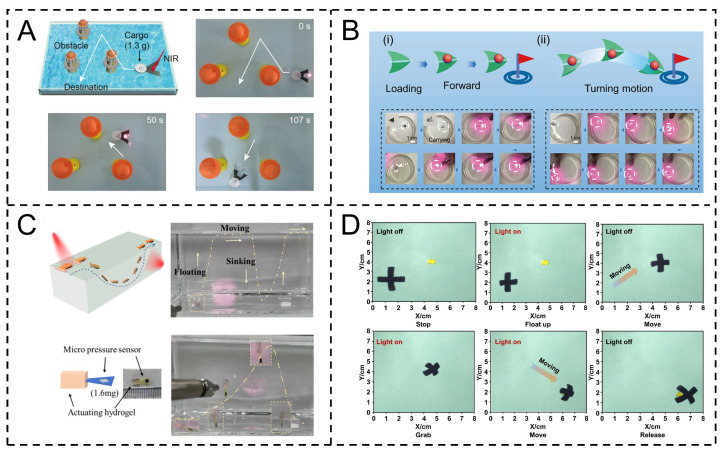
Application of goods transportation mediated by the Marangoni effect. (**A**) High-load transportation with near-infrared photothermal regulation. Reproduced from Reference [106] with permission from *ACS Applied Materials & Interfaces*. (**B**) A circulating transport system for heat-responsive hydrogels. Reproduced from Reference [107] with permission from Small. (**C**) Transportation of oil-phase goods with hydrophobic foam. Reproduced from Reference [108] with permission from *ACS Applied Materials & Interfaces*. (**D**) Full-process transportation of light-controlled deformed grippers. Reproduced from Reference [109] with permission from the *International Journal of Extreme Manufacturing*.

For instance, Xiang et al. achieved controllable liquid–gas interface movement via NIR-induced photothermal effects and Marangoni gradients. Their device transports cargo weighing approximately 30 times its own weight (such as buttons), with NIR-adjustable trajectories and obstacle avoidance (Figure 10A) [106]. Chen et al.’s hydrophobic PVDF/Ti_3_C_2_T_x_ porous foam photothermal actuator enables non-aqueous solution cargo transportation. Via Ti_3_C_2_T_x_’s photothermal conversion and PVDF’s lipophilicity, infrared-induced gradients drive multi-mode motion (up to 6 mm/s), achieving fixed-point delivery, path following, and obstacle avoidance for micro-cargo in oil systems (Figure 10B) [107].

In addition, Zhang et al. prepared PNIPAM hydrogels with PDA-HGMPs (polydopamine-modified hollow glass microspheres, efficient photothermal conversion, low density). NIR regulation enables reversible floating/sinking via thermal-responsive density changes, achieving “float-move-sink” motion (5–17 mm/s) with adjustable direction and stable cycling (>20 cycles). It precisely delivers light loads and integrates with sensors for transport–perception integration, facilitating micro-actuator cargo transportation (Figure 10C) [108]. Zhan et al. fabricated NIPAM-based, carbon-nanoparticle-modified constructs via 3D printing, responsive to NIR. Adjusting light enables rapid reversible deformation (1–5 s). Asymmetric micro-grippers contract to grasp objects (max 4.8 N) under NIR, releasing when light is off. The Marangoni effect from asymmetric temperature gradients allows speed (up to 1 mm/s) and direction control, completing grasp–float–move–release for aqueous micro-cargo transport (Figure 10D) [109].

#### 4.2.3. Programmable Motion Control

In research on programmable motion control, the spatiotemporal response characteristics of the Marangoni effect have been applied to design bionic motion units. By programmatically regulating the evolution law of the interfacial tension field, the coordinated motion mode of biological groups can be simulated, providing a new paradigm for the control of intelligent robot clusters and the development of flexible mechanical systems.

**Figure 11:**
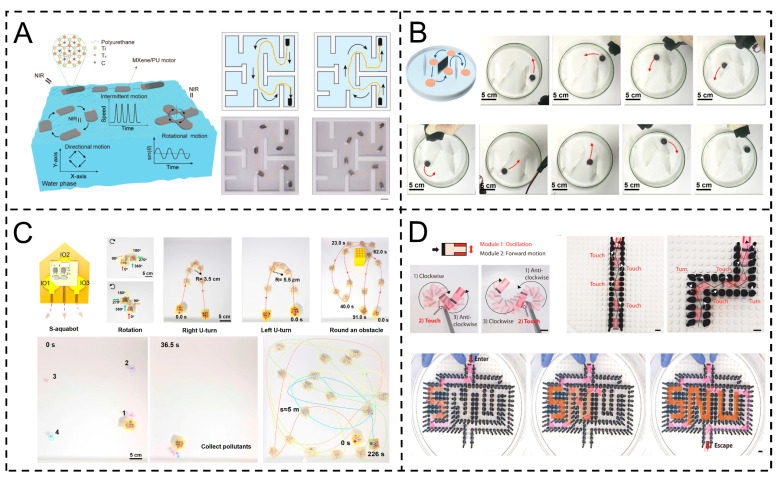
Programmable motion control application mediated by the Marangoni effect. (**A**) Complex spatial motion programming for photothermal regulation. Reproduced from Reference [110] with permission from the *Chemical Engineering Journal*. (**B**) Path planning and obstacle avoidance driven by light. Reproduced from Reference [111] with permission from *Sensors and Actuators B: Chemical*. (**C**) Precise control of multi-mode trajectory through electronic control. Reproduced from Reference [61] with permission from *eScience*. (**D**) Complex action programming with modular collaboration. Reproduced from Reference [112] with permission from *Nature Communications*.

Xu et al.’s MXene/PU motor, with a “brick-mortar” structure, achieves programmable motions via the NIR-driven photothermal Marangoni effect: directional movement (light-position-controlled), rotation (edge-light-point-adjusted), and intermittent motion (light ON/OFF). Speed correlates with laser power; it navigates mazes precisely, enabling complex spatial movement (Figure 11A) [110]. Jing et al.’s PAAm/SA (sodium alginate)–GE (graphene oxide) driver, leveraging GE’s photothermal conversion, achieves programmable motions (directional linear movement, flexible turning with >180° rotation, intermittent motion) via infrared-laser-adjusted irradiation points, driven by photothermal-induced surface tension gradients (Marangoni effect). It navigates an “N”-shaped maze within 2 min, demonstrating complex space obstacle avoidance and preset route following (Figure 11B) [111].

In addition, Zhou et al.’s S-aquabots, based on PM-motors, achieve programmable motion via flexible hybrid electronics-controlled ethanol release and Marangoni gradients. Three channels enable forward movement (max 18 cm/s), rotation (360° in 5.5–7.5 s), turning (right turning radius of 3.5 cm, left turning radius of 5.5 cm), obstacle bypassing, and pollutant collection, demonstrating precise trajectory control (Figure 11C) [61]. Song et al. designed a pen-drawn Marangoni actuator, achieving complex motion planning via combined functional modules (oscillation, rotation, intermittent motion, linear advancement). Head oscillation combined with tail propulsion enables maze traversal (“SNU” shape); green (counterclockwise rotation) and red (clockwise rotation) module combinations realize collaborative actions like underwater robot football dribbling/shooting, demonstrating module-based programmable motion (Figure 11D) [112].

#### 4.2.4. Other Applications

**Figure 12:**
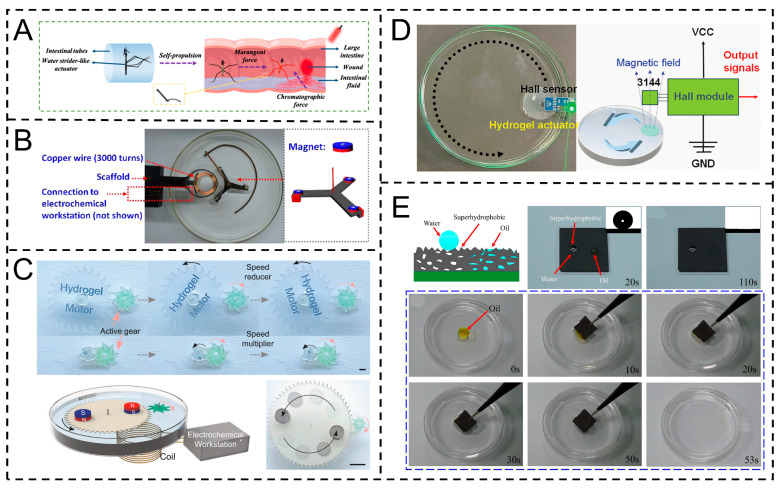
Other extended applications of the Marangoni effect. (**A**) Intestinal targeted drug delivery system. Reproduced from Reference [113] with permission from *Materials Today Bio*. (**B**) Electromagnetic induction power generation empowered by supramolecular strategies. Reproduced from Reference [88] with permission from *CCS Chemistry*. (**C**) Long-endurance hydrogel rotor power generation system. Reproduced from Reference [114] with permission from *Nature Communication*. (**D**) Wireless sensing. Reproduced from Reference [115] with permission from *Advanced Materials*. (**E**) Environmental cleanliness of superhydrophobic composite materials. Reproduced from Reference [116] with permission from *Chemical Engineering Journal*.

In the biomedical field, by leveraging its precise control over biocompatible interfaces, the Marangoni actuator can achieve targeted delivery and site-specific release of drug carriers in complex physiological environments. By responding to chemical signals in the lesion area (such as pH value), it triggers interfacial tension mutations, thereby significantly enhancing drug delivery efficiency and treatment accuracy. Inspired by water striders, Zhang et al.’s intestinal scaffold actuator, with a core Janus sticky hydrogel patch, enables targeted drug delivery in complex physiological environments. Its superhydrophilic layer (reverse opal structures) responds to intestinal pH for positioning; Cu-containing legs convert IR to heat to regulate intestinal wall adhesion. It binds via hydrogen bonds, moves directionally via intestinal pH gradients and the Marangoni effect, transports drugs unidirectionally via capillary force, is excretable naturally, and shows 20-day stability (Figure 12A) [113].

In the field of energy conversion, the Marangoni actuator can convert the interfacial energy gradient into mechanical energy by driving the periodic movement of droplets on a specific structural surface and then couple piezoelectric or triboelectric components to achieve continuous power generation, providing an innovative idea for the development of micro-energy devices. For instance, Cheng et al. extended Marangoni movement duration via supramolecular strategies for power generation devices. Based on electromagnetic induction, magnets on three wings rotate above 3000-turn coils, inducing current (max 0.7 mV) through magnetic flux changes, converting interfacial tension gradient energy into electricity (Figure 12B) [88]. Similarly, Wu et al.’s Marangoni hydrogel rotor excels in power generation via electromagnetic induction; it drives a magnet-integrated passive rotor to rotate, inducing voltage through magnetic flux changes. With 5215 rpm rotation and 34.6 min fuel economy, it reaches 740 mV, boosted to 3.4 V to light LEDs, operating stably over 8 min (Figure 12C) [114].

In addition, in the field of sensing applications, based on the Marangoni effect (the surface tension gradient caused by the solvent exchange between ethanol and water), ethanol-containing polyelectrolyte hydrogel actuators can exhibit shaped-dependent regular movements. This characteristic and stable signal output capability provide the possibility for the construction of new wireless sensing devices. Yin et al.’s annular device with a hydrogel actuator (loaded with a neodymium magnet) performs stable, repeatable circular motion (>50 times, >17 min) on water via the Marangoni effect. The magnet periodically triggers a Hall sensor, generating stable 0–5 V digital signals (42 times/5 min) for high-precision sensing (Figure 12D) [115]. In the field of environmental cleaning, based on the dual characteristics of superhydrophobicity and lipophilicity of the actuator superhydrophobic composite material, it is possible to achieve efficient enrichment of oil stains and microplastic pollutants in water bodies. Wang et al.’s graphene/PDMS superhydrophobic composite (water contact angle > 150°) with micro-nano structures exhibits superhydrophobicity and lipophilicity. It selectively adsorbs surface oil, removes water-insoluble pollutants via adhesion, eliminates water-soluble ones via dissolution, and restores cleanliness after water washing (Figure 12E) [116].

Notably, amid the diverse cross-scale applications outlined above, a clear distinction emerges between technologies that have advanced toward practical maturity and those remaining in early developmental stages. Microfluidic manipulation, validated in lab-scale systems for droplet sorting and particle transport with clear integration pathways into microfluidic chips, and environmental cleanup via superhydrophobic composites, which have demonstrated consistent performance in pilot-scale water treatment with established material recycling protocols, represent relatively advanced stages with scalable routes. In contrast, programmable swarm behaviors, constrained by trajectory synchronization issues in dynamic environments, and in vivo biomedical delivery systems like pH-responsive intestinal scaffolds, requiring optimization of biocompatibility and degradation kinetics, remain in early developmental phases. This differentiation not only contextualizes the current technological landscape but also underscores the need for targeted innovations to address bottlenecks in emerging applications, aligning with the earlier analysis of mechanism-specific challenges in material and structural design.

## 5. Summary and Perspectives

This paper systematically reviews the research progress on Marangoni actuators and elaborates in depth their core driving mechanism, material structure design, driving mode, and multi-dimensional applications.

At the principle level, it is clear that the Marangoni effect originates from the interfacial tension gradient, mainly divided into two triggering mechanisms: temperature gradient and solute concentration gradient. Based on this, the material selection logic of the actuator is analyzed. The temperature-gradient-driven type focuses on the high-performance characteristics of photothermal conversion materials, while the solute-concentration-gradient-driven type relies on the controllable release performance of surfactants. At the same time, it emphasizes the crucial role of an asymmetric actuator structure in achieving directed motion. In terms of driving methods, it covers multiple external field control modes, such as light drive (regulating temperature gradient based on photothermal effect), chemical-driven (regulating solute concentration gradient through alcohol, pH value, SDS, etc.), and electric drive (modulating interfacial tension distribution through electrical signals), demonstrating the differentiated characteristics of each driving method in response rate, controllability, and environmental adaptability. At the application level, this type of actuator demonstrates cross-scale application potential; in the microscopic field, it can achieve salt ion transport, bubble manipulation, energy conversion, and regulation of biological group behavior. In the macro field, it demonstrates unique value in scenarios like supramolecular assembly, cargo transportation, programmable motion control, biomedical targeted drug delivery, energy conversion, environmental cleaning, and sensor detection. Overall, the Marangoni actuator, through its precise control of the interfacial tension gradient, provides a new technical path for the development of intelligent micro-nano devices and flexible mechanical systems. Its research achievements are of great significance for promoting the development of fields like microfluidic technology, robotics, and biomedical engineering.

In the future, intelligent actuators based on the Marangoni effect, as a type of advanced functional device that achieves precise control relying on interfacial tension gradients, will demonstrate broad application prospects and development potential:

(1) The invention of high-performance functional materials will provide core support for the performance leap of Marangoni actuators. For instance, the continuous optimization of new photothermal conversion materials (such as MXene, black phosphorus, etc.) can further enhance the efficiency of light energy–thermal conversion, improve the accuracy of temperature gradient regulation, and thereby increase the movement speed and response sensitivity of the actuator. The combination of stimuli-responsive intelligent polymers and surfactants will endow the actuator with more efficient solute release kinetic regulation capabilities, extend its motion life, and expand its environmental adaptability.
(2) The development of multi-field collaborative drive and precise programming technology will be the key direction for its intelligent upgrade. The limitations of a single optical drive or chemical-driven or electric drive will gradually be broken through. Through the collaborative coupling of multiple physical fields, such as light–electricity and chemical–thermal, diverse combinations of actuator motion modes and precise planning of complex trajectories can be achieved. For instance, by integrating light-controlled navigation with solute release triggered by electrical signals, it is expected to achieve intelligent execution of “motion-operation” integration, laying the foundation for cluster collaboration in complex scenarios.
(3) The enhancement of functional integration and environmental adaptability will expand its application boundaries. In the future, Marangoni actuators will not only have self-propulsion capabilities but also integrate micro-sensing units (such as pH, temperature, and pollutant sensors) to achieve real-time perception and dynamic response to environmental signals. This feature is particularly important in the biomedical field. For instance, actuators sensitive to the microenvironment of the lesion (such as acidic pH) can be designed to achieve targeted drug delivery and on-demand release, significantly enhancing the accuracy of treatment. In environmental monitoring, in situ detection and autonomous collection of water pollutants can be achieved.
(4) Deep penetration in both micro-manipulation and macro-application scenarios will become an important development trend. At the micro level, its application in precise directional transport in microfluidic chips, capture and assembly of cells/particles, and other fields will become more mature, providing efficient tools for the preparation of biochips and micro-nano manufacturing. At the macro level, bionic swimming pools and environmental cleaning robots based on the Marangoni effect will play a significant role in tasks like cleaning oil stains on water surfaces, collecting water pollutants, and even programmable assembly of macroscopic supramolecular materials, demonstrating cross-scale application value from micro to macro.
(5) Breakthroughs in low-cost and large-scale manufacturing processes will accelerate its industrialization process. The current preparation mode that relies on complex processes, such as precision lithography and laser processing, will gradually develop towards simplification and high-throughput. For instance, the introduction of technologies like template-assisted printing and 3D printing can enable rapid customized production of actuators, reduce manufacturing costs, and provide the possibility for their popularization in large-scale application scenarios such as environmental governance and flexible robots.
(6) Advancement of theoretical modeling for complex fluid systems: The accurate prediction of Marangoni flow dynamics in non-ideal environments remains a critical bottleneck. There is an urgent need to develop multi-physics theoretical frameworks that integrate viscoelastic properties of biological fluids, phase interactions in multiphase emulsions, and dynamic interfacial evolution. Such models will enable quantitative simulation of actuation behaviors under realistic working conditions (e.g., in vivo microenvironments or industrial complex flow fields), providing mechanistic insights for rational material design and performance optimization.

In conclusion, with the continuous advancement of materials science, drive regulation, and integration technology, intelligent actuators based on the Marangoni effect are expected to achieve breakthroughs in many fields, such as micro-nano manipulation, biomedicine, and environmental engineering. Their unique interfacial tension driving mechanism and programmable characteristics are likely to become one of the core technologies for solving micro-scale precision operations and complex environmental adaptability tasks, making significant contributions to the technological progress and sustainable development of human society.

## Figures and Tables

**Table 1:** Representative gel-based and composite Marangoni actuators: material compositions, trigger mechanisms, and performance characteristics.

Representative Marangoni Actuators	Material Composition	Trigger Mechanism	Key Performance Metrics	Reference
PDMS/Fe_3_O_4_@SA bilayer photothermal actuator	PDMS/Fe_3_O_4_ composite (PIC layer) + sodium alginate (SA) film	Photothermal effect (Fe_3_O_4_-mediated light-to-heat conversion) → temperature gradient	Efficient photothermal conversion; enables directional motion via surface tension gradients	[49]
BP-enhanced PVDF microfiber membrane actuator	PVDF microfiber membranes (MFF) + black phosphorus (BP) + copper–nickel conductive fabrics	Photothermal effect (BP-mediated light-to-heat conversion) → temperature gradient	Enhanced photothermal conversion efficiency; controllable motion on water surface	[12]
Candle soot (CS)/PDMS composite actuator	Candle soot (CS) + PDMS	Photothermal effect (CS-mediated light-to-heat conversion) → temperature gradient	Efficient light absorption and heat generation; stable surface tension gradient formation	[50]
CuS-doped PNIPAM hydrogel actuator	PNIPAM hydrogel doped with CuS nanoparticles	Photothermal effect (CuS-mediated light-to-heat conversion) → temperature gradient	Near-infrared responsiveness; programmable motion trajectories	[52]
HFIP/SRT protein matrix actuator	Hexafluoroisopropanol (HFIP) + SRT protein matrix	SRT protein’s dynamic nanostructural changes → controllable HFIP release → concentration gradient	Self-regulated solute release; stable surface tension gradient maintenance	[54,56]
PVA fuel-mediated actuator	Polyvinyl alcohol (PVA) loaded in PEGDA hydrogel + hydrophobic polyurethane acrylate	Controlled release of PVA → concentration gradient	Biodegradable fuel; adjustable surface tension gradient	[55]

**Table 2:** Representative gel-based Marangoni actuators.

Gel Type	Speed (mm/s)	Endurance	Payload Capacity	Path Accuracy	Reference
Oxazine Hydrogel	Up to 14.53	Hours	Not specified	High (programmable)	[65]
MXene-Chitosan Hydrogel	Initial: 18.3	50 min	Cargo transport (rubber)	High (maze navigation)	[19]
Fe^3+^/Catechol Gel	5.0 (3.5 cm/7 s)	>20 min	2× body weight	High (obstacle avoidance)	[66]
PNIPAM-CuS Hydrogel	Up to 43	>50 cycles	Plastic ball transport	High (3D trajectory)	[52]
Au-PNIPAM Hybrid Gel	2.5	>10 cycles	Not specified	Moderate (linear/rotational)	[67]

**Table 3:** Benchmark performance metrics of representative Marangoni actuators.

Actuator System	Max Velocity (mm/s)	Response Time (s)	Minimum Laser Power/Irradiance	Fuel Consumption Rate	Motion Lifetime	Turning Radius (mm)	Controllability
PDMS/Fe_3_O_4_@SA bilayer photothermal actuator (Figure 3A)	8.2	1.2	1.5 W/cm^2^ (808 nm)	N/A (photothermal)	>2 h (continuous irradiation)	15 ± 2	On/off (laser toggle); programmable via irradiation position
BP-enhanced PVDF microfiber membrane actuator (Figure 3B)	12.5	0.8	0.8 W/cm^2^ (980 nm)	N/A (photothermal)	>1.5 h (continuous irradiation)	10 ± 3	On/off (laser toggle); directional control via edge irradiation
CS/PDMS composite actuator (Figure 3C)	6.7	1.5	2.0 W/cm^2^ (808 nm)	N/A (photothermal)	>3 h (continuous irradiation)	20 ± 4	On/off (laser toggle); limited programmability
CuS-doped PNIPAM hydrogel actuator (Figure 3E and Figure 5B)	43.0	0.5	0.45 W/cm^2^ (808 nm)	N/A (photothermal)	>50 cycles (heating–cooling)	8 ± 2	On/off (laser toggle); 3D trajectory programmability (ascend/descend/translate)
SDS-driven multi-engine rotor (Figure 6E)	9.3	5.0	N/A (chemical)	0.02 mg/s (SDS)	2400 s (β-CD regulated)	12 ± 3	On/off (fuel depletion/supplementation); rotational speed tunable

## Data Availability

No new data were created or analyzed in this study. Data sharing is not applicable to this article.
